# To: First Brazilian recommendation on physiotherapy with sensory motor stimulation in newborns and infants in the intensive care unit

**DOI:** 10.5935/0103-507X.20220032-en

**Published:** 2022

**Authors:** Sheila Tamanini de Almeida, Deborah Salle Levy, Carla Lucchi Pagliaro, Carolina Castelli Silvério

**Affiliations:** 1 Department of Speech Therapy, Universidade Federal de Ciências da Saúde de Porto Alegre - Porto Alegre (RS), Brazil.; 2 Department of Health and Human Communication, Instituto de Psicologia, Universidade Federal do Rio Grande do Sul - Porto Alegre (RS), Brazil.; 3 Centro de Pronto Atendimento Pediátrico - São Paulo (SP), Brazil.; 4 Department of Disfhagia, Sociedade Brasileira de Fonoaudiologia/Gestão 2020-22 - São Paulo (SP), Brazil.

**TO THE EDITOR**

The special article presented by Johnston et al., entitled “First Brazilian recommendation of physical therapy for sensorimotor stimulation of newborns and infants in an intensive care unit”,^(1)^ aimed to provide guidelines for sensorimotor stimulation. However, caution should be used when analyzing and interpreting the results of the included studies and developing guidelines for the recommendations.

To recommend the modalities of therapeutic massage, i.e., tactile-kinesthetic stimulation and multisensory stimulation to improve sucking, the results were aggregated for two different outcomes: weight and sucking. For a better interpretation of the results of the studies, it should be noted in tables 5S, 6S and 8S of the commented article that only in the latter, there is a a single publication that evaluated suction.^(2)^ According to the analysis of the authors of this recommendation, the study has a moderate level of evidence, which we agree with. However, the results and conclusions should be interpreted with caution due to the limitations of the study, as noted by the authors themselves: the small sample size; the heterogeneous sample of infants with and without medical complications; and the lack of daily collection of data on sucking, which hinders the understanding of the variability of daily behaviors. All these findings limit the ability to generalize a recommendation. When analyzing the multisensory approach, this study did not control for confounding factors, thus leading to a risk of measurement bias. Therefore, there is no way to isolate the effect of multisensory stimulation as the only factor favoring the sucking performance of newborns subjected to auditory, tactile, visual and vestibular stimulation.^(2)^

The authors of this document, which integrates and represents the 20202022 management of the Department of Dysphagia of the *Sociedade Brasileira de Fonoaudiologia* (SBFa), also highlight the role of the speech therapist as responsible and qualified for prevention, evaluation, diagnosis, functional/functional rehabilitation and management of sucking and swallowing disorders, acting in units of low, medium and high complexity, hospitals (including neonatal intensive care units), rehabilitation centers, among others.^(3)^ Thus, specialized literature in this area may provide more robust evidence focused on the functions of sucking, swallowing and feeding.

For example, a double-blinded randomized clinical trial using an oral stimulation program (applied by speech therapists) before the first attempt at oral assessment showed promising results. The intervention group reached level 4 of oral feeding ability 8 days before the control group, and 75.7% of participants reached level 4in the first oral attempt. The control group had a lower probability of reaching 100% of the diet when compared to the intervention in the same period (p = 0.024), and only 16.2% of the newborns in the first group reached skill level 4, performing the transition from feeding to oral route twice as long.^(4)^

In addition, the Cochrane Review highlighted the benefits of oral stimulation in accelerating the transition to full feeding, reducing the length of hospital stay and duration of feeding, with no significant difference in breastfeeding and weight gain.^(5)^

The relevance of recommendations such as the one published in this journal is emphasized, but they should be carefully reviewed so that the possible effects of multisensory stimulation are not generalized to generate confounding factors and nonspecific conclusions. It also points to the importance of scientifically directing each area of stimulation to the qualified professional. Intraoral stimuli, such as sucking, can be applied to promote intervention objectives in different areas; however, prevention, evaluation, diagnosis, functional suction/rehabilitation and swallowing are affected by the competence of the speech-language pathologist.
